# Cerebrospinal fluid-cutaneous fistula associated with post-traumatic Charcot spinal arthropathy: a case report and review of literature

**DOI:** 10.1186/s12891-020-03451-7

**Published:** 2020-06-29

**Authors:** Ji Hyun Ryu, Jun-Seok Lee, Chang-Rack Lim, Wan Jae Cho, Ki-Won Kim

**Affiliations:** 1 Department of Orthopaedic Surgery, Yeouido St. Mary’s Hospital, College of Medicine, The Catholic University of Korea, 63-ro 10 Yeongdeungpo-gu, Seoul, 07345 South Korea; 2 Department of Orthopaedic Surgery, Eunpyeong St. Mary’s Hospital, College of Medicine, The Catholic University of Korea, Tongil-ro 1021 Eunpyeong-gu, Seoul, South Korea

**Keywords:** Charcot spinal arthropathy, CSF-cutaneous fistula, Spinal cord injury, Four-rod spinopelvic fixation

## Abstract

**Background:**

Charcot spinal arthropathy, also known as Charcot spine and neuropathic spinal arthropathy, is a progressive and destructive condition that affects an intervertebral disc and the adjacent vertebral bodies following loss of spinal joint innervation. We report the first case of Charcot spinal arthropathy (CSA) associated with cerebrospinal fluid (CSF)-cutaneous fistula.

**Case presentation:**

A 54-year-old male who underwent T10-L2 posterior instrumented spinal fusion seven years prior for treatment of T11 burst fracture and accompanying T11 complete paraplegia visited our department complaining of leakage of clear fluid at his lower back. The patient had also undergone various types of skin graft and myocutaneous flap surgeries for treatment of repetitive pressure sores around his lumbosacral area. The patient presented with persistent CSF leakage from a cutaneous fistula (CSF-cutaneous fistula) formed in a lumbosacral pressure sore. The CSF-cutaneous fistula arose from the L5 post-traumatic CSA. Surgery was planned for management of CSF-cutaneous fistula and post-traumatic L5 CSA. We successfully treated the CSF-cutaneous fistula with ligation and transection of the dural sac and cauda equina at the L2-L3 level. In addition, the post-traumatic L5 CSA was successfully treated with a posterior four-rod spinopelvic fixation from T9 to ilium and S2 foramina. After surgery, the CSF leakage stopped and no other adverse neurological changes were found. The four-rod spinopelvic construct was well maintained five years later.

**Conclusions:**

CSA associated with CSF-cutaneous fistula is a very rare disorder. Only surgical treatment for both CSA and CSF-cutaneous fistula with ligation and transection of the dural sac and posterior four-rod spinopelvic fixation can bring satisfactory results.

## Background

Charcot spinal arthropathy (CSA), also known as Charcot spine and neuropathic spinal arthropathy, is a progressive and destructive condition that affects an intervertebral disc and the adjacent vertebral bodies following loss of spinal joint innervation for any reason [1, 2]. The first case of CSA associated with tabes dorsalis (non-traumatic CSA) was reported in 1884 by Kronig [3]. Approximately 90 years later, the first case of post-traumatic CSA was reported in 1978 by Slabauch et al. [4]. Currently, with advances in medical care, traumatic spinal cord injuries are the leading cause of CSA [2, 5]. Between 1978 and 2018, over 140 post-traumatic CSA cases have been reported in the literature [2, 6–24].

Until mid-twentieth century, patients sustaining catastrophic traumatic spinal cord injury might have died of injury-related complications [5, 25, 26]. However, with recent advances in medical care, spinal cord injured patients are now leading full and productive lives [5]. The improvement in their care and longevity makes the traumatic spinal cord injury the leading cause of CSA in recent times [2, 5].

Irrespective of non-traumatic or post-traumatic CSA, considerable advances in spinal surgery techniques and technology during the past few decades have altered treatment paradigms from conservative management to surgery [6, 13, 27]. In cases of CSA involving gross spinal instability and absence of medical comorbidities that would otherwise cause a contraindication, surgery has become the preferred treatment modality [6, 13, 27]. Most of the recent literature recommends a combined anterior-posterior circumferential fusion through a single-stage or multi-staged approach to reduce the rate of hardware failure [27]. Alternatively, single-stage, posterior three-column resection shortening and fusion may avoid the potential complications of a long anterior cage or an allograft segment [28]. Four-rod lumbopelvic fixation particularly in lumbar CSA and bone morphogenetic protein were also recommended to reduce hardware failures [13].

Because of its slow progression and sensory denervation, diagnosis of post-traumatic CSA is delayed and presents as a late complication of traumatic spinal cord injury [2, 6, 29].

We report a post-traumatic CSA associated with CSF-cutaneous fistula case of a male patient treated with ligation and transection of the dural sac and posterior four-rod spinopelvic fixation.

## Case presentation

Consultation was requested from the department of plastic surgery for a 54-year-old male who developed leakage of clear fluid in a lumbosacral pressure sore. He had previously suffered T11 burst fracture and resultant complete paraplegia in a motorcycle accident seven years prior. At the time of the injury, he had received a T9-L2 posterior instrumented spinal fusion at another hospital. Being in a paraplegic state, pressure sore soon developed thereafter. The patient also underwent various types of skin graft and myocutaneous flap surgeries to treat for the repetitive pressure sores around his lumbosacral area. On physical exam, clear, colorless, tap water-like fluid leaked from a small, deep, fibrotic opening formed at a lumbosacral pressure sore. Based on the nature of the fluid and the depth of the opening, we suspected that the liquid from the persistent leakage might be CSF. In addition, although completely paraplegic, the patient reported a sense of lumbosacral instability and kyphosis when in a sitting position and he heard cracking noises in his lumbosacral region upon movement. These symptoms were not associated with pain.

**Fig. 1:**
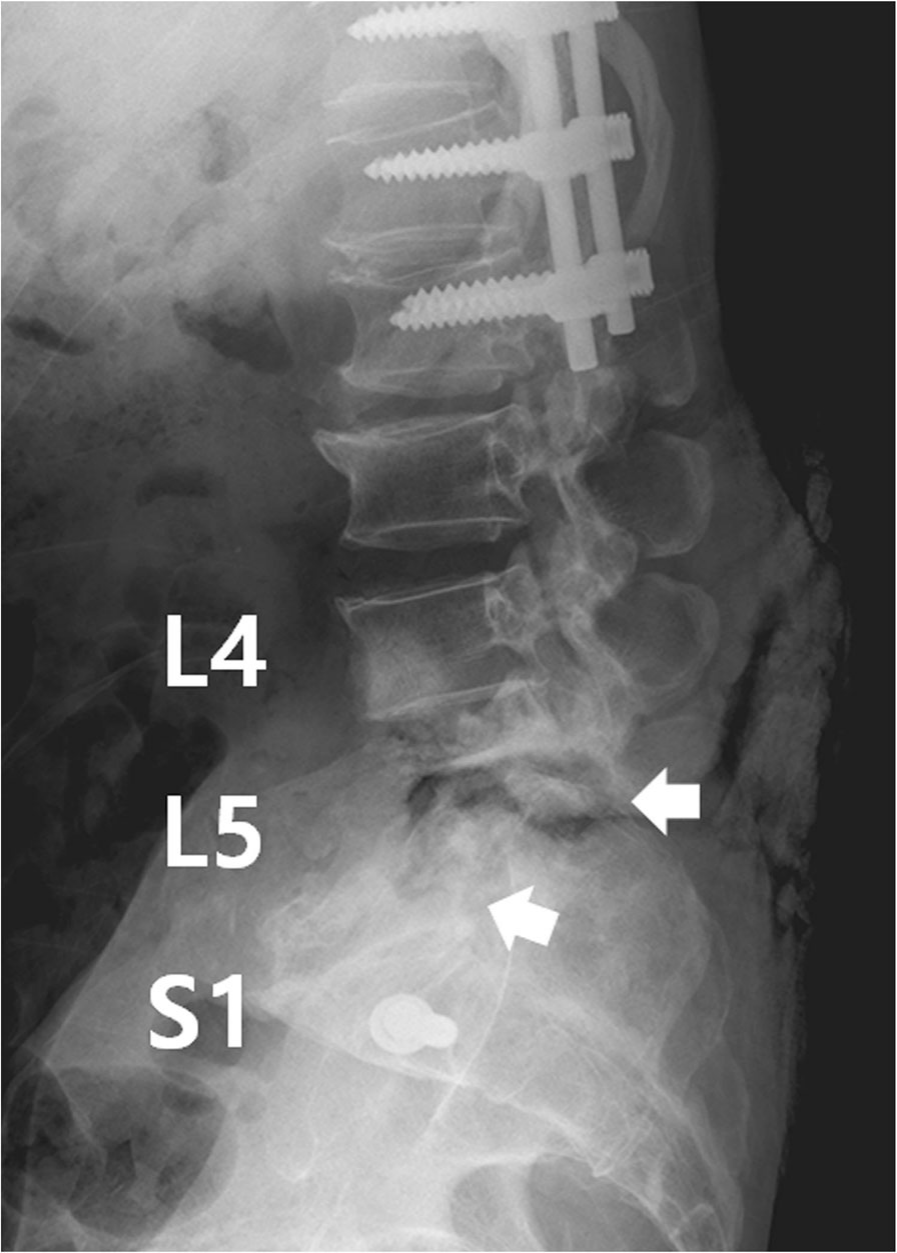
Radiography of the lumbar spine before surgery shows horizontal split of the L5 vertebral body and complete posterior dissociation of the upper spinal segment with respect to the lower one through the split site (white arrow)

**Fig. 2:**
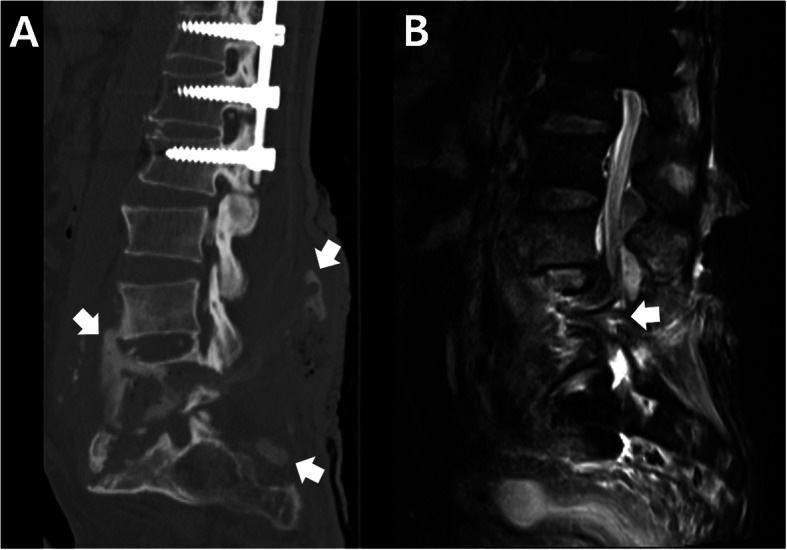
**a** CT shows extensive destruction of the L5 vertebral body and relatively intact endplates. Myelogram dye injected at the L2-L3 level leaked through a fistula from the L5 CSA region to a lumbosacral pressure sore (white arrows). **b**. MRI shows complete transection of the dural sac and cauda equina (white arrow) by destructed bony fragments and surrounding fibrotic tissues

Lateral plain radiograph showed a horizontal split of the L5 vertebral body and complete posterior dissociation of the upper spinal segment with respect to the lower one (Fig. 1). Computed tomography (CT) myelogram showed an extensive destruction of the L5 vertebral body and relatively intact superior and inferior endplates. The myelogram dye injected at the L2-L3 level leaked through a fistula from the L5 CSA region to the lumbosacral pressure sore (Fig. 2a). This finding confirmed that the clear, colorless fluid was CSF and its continuous flow was caused by a CSF-cutaneous fistula [30]. Fortunately, our patient had no apparent symptoms related to the CSF-cutaneous fistula. Magnetic resonance imaging (MRI) revealed complete transection of the dural sac and cauda equina by destructed bony fragments and surrounding fibrotic tissues (Fig. 2b). These radiographic findings were consistent with those of previously reported CSA [2, 31]. Consequently, we established the diagnosis of post-traumatic CSA associated with CSF-cutaneous fistula.

**Fig. 3:**
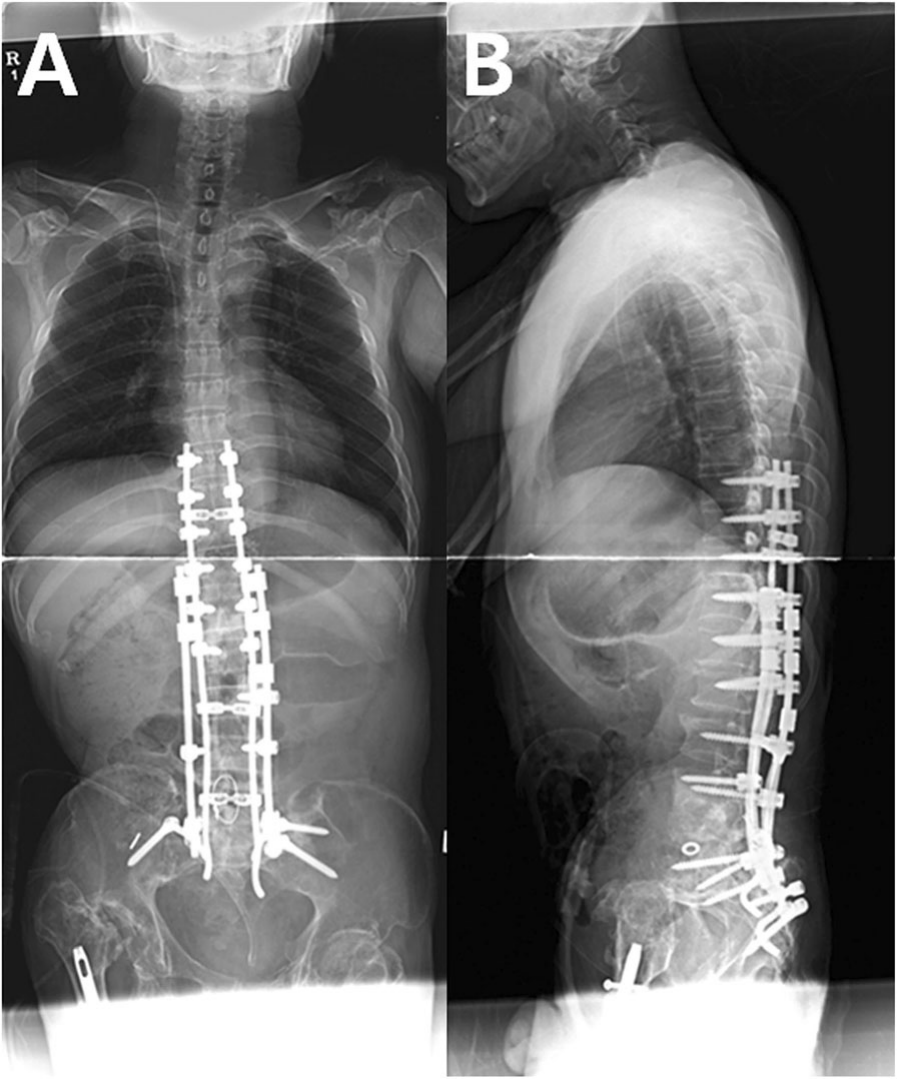
Anteroposterior(**a**) and lateral(**b**) whole spine radiography after surgery shows posterior four-rod spinopelvic fixation

**Fig. 4:**
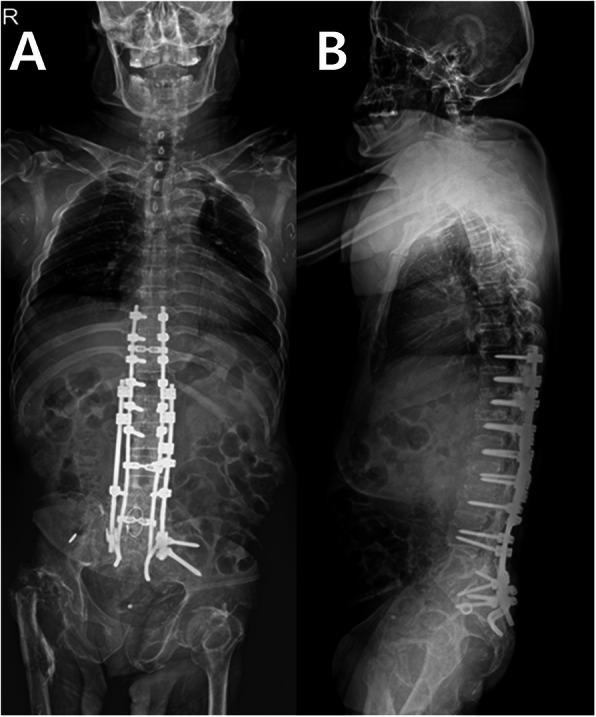
Anteroposterior(**a**) and lateral(**b**) whole spine radiography 5 years after surgery. A right iliac screw was removed at postoperative one year because of skin irritation

Surgery was planned for management of post-traumatic L5 CSA and CSF-cutaneous fistula. After dissecting from T8 to the ilium, laminectomy was performed at the L2-L3 level and the exposed dural sac was doubly ligated circumferentially with 3–0 black silk. The dural sac and cauda equina just distal to the double-ligation site were transected completely with a surgical blade. The absence of CSF leakage from the proximal cutting site was confirmed with the Valsalva maneuver. Next, the patient underwent posterior four-rod spinopelvic fixation from T9 to the ilium and S2 foramina. Two long rods were inserted into the S2 foramina and the posteriorly dissociated upper spinal segment was reduced with a cantilever maneuver. These two rods were fixed to the intermittent lumbar and thoracic pedicle screws. Then, two short rods were fixed to iliac screws and intermittent lumbar pedicle screws. Each pair of long and short rods on the right and left sides was attached to each other with side-connectors (Fig. 3a, b). Culture study performed during surgery revealed *Pseudomonas aeruginosa* and methicillin-resistant *Staphylococcus aureus*. After surgery, vancomycin and imipenem were administered to the patient to cover the two bacterial organisms. The CSF leakage stopped and no adverse neurological changes were found. One iliac screw inserted into the right ilium was removed at postoperative one year due to skin protrusion. The four-rod spinopelvic construct was well maintained five years later (Fig. 4a, b).

## Discussion and conclusions

Loss of protective sensory innervations, such as proprioception and sensitivity to pain and temperature, is the main disease mechanism in CSA [2]. The time lag between onset of neurological impairment and diagnosis of CSA is usually long (over 17 years, on average). The reasons for this long delay are likely due to the characteristics slow progression of CSA pathology and the relatively non-specific nature of apparent symptoms [2, 13]. Post-traumatic CSA only develops caudal to the neurological level of injury, which is consistent with the proposed mechanism of CSA [7, 13]. Post-traumatic CSA is more common in patients with complete paraplegia who are actively mobilized, especially in wheelchairs, and have significant weight-bearing in sitting positions [7, 13]. Most post-traumatic CSAs occur within the lumbar spine, where increased lumbar mobility may predispose to excessive forces during self-transfer activity [13]. Particularly, in patients who previously underwent instrumented fusion for treatment of spine fracture and neurologic impairment, post-traumatic CSA typically develops below the prior instrumented spinal fusion area [2, 13]. These notions proposed by the authors of large studies are further supported by the patient in the present case, in whom post-traumatic CSA occurred at the L5 vertebra which is three vertebral levels caudal to the end of the prior instrumented fusion.

The two major symptoms of CSA are spinal deformity/instability in sitting position and an audible cracking noise with movement [2]. Hypertrophic osteophytes with disc and vertebral destruction are radiologic key findings of CSA [2, 6]. These changes induce instability and dislocation of the affected level. Furthermore, scoliosis, kyphosis or combined spinal deformity can occur at any part of the spine [1, 2]. The patient in the present case had a seven-year history of complete paraplegia, along with the two major symptoms of CSA. Also, the patient had dislocation and instability at dynamic x-ray. Because the spinal deformity/instability and cracking noise in our patient were not associated with pain, the diagnosis of post-traumatic CSA had been delayed until leakage of CSF from the CSF-cutaneous fistula formed at a lumbosacral pressure sore.

Our patient’s unique problem that has not been previously reported in the literature is a CSF-cutaneous fistula associated with CSA. The CSF-cutaneous fistula arose from the L5 CSA level and was confirmed by CT myelogram. CT myelogram and MRI revealed complete transection of the dural sac and cauda equina by destructed bony fragments and fibrotic tissues. Dynamic instability-induced repetitive traction and compression of the dural sac by the tissues at the level of the post-traumatic CSA might have progressively weakened and torn the dural sac. However, a dural tear itself may not lead to CSF leakage if the spinal muscles and skin overlying the tear are intact and exert a sufficient tamponade effect. We assume that the multiple flap surgeries for treatment of our patient’s lumbosacral pressure sores led to progressive weakening and disruption of the myocutaneous tamponade effect, consequently leading to CSF leakage. Persistent CSF leakage, if managed improperly, leads to a CSF-cutaneous fistula [30]. CSF-cutaneous fistula is rare but can cause serious complications, such as intracranial hemorrhage, bacterial meningitis and cranial nerve palsy [30, 32–34].

Although our patient had no apparent symptom related to the CSF-cutaneous fistula, our primary concern was its management. Because our patient had T11 complete paraplegia and the damage to the dural sac and cauda equina appeared to be extensive on MRI, we thought that repairing the damaged dural sac and saving the cauda equina were unnecessary. To prevent potential complications related to CSF-cutaneous fistula, we ligated and transected the dural sac and cauda equina at the L2-L3 level, which was below the neurological level of his T11 complete paraplegia and above the level(L5) of the origin of the CSF-cutaneous fistula.

Because of the low incidence of CSA, it has been difficult to establish a consensus as to its optimal treatment [13]. However, considerable advances in spinal surgery techniques have altered the treatment paradigms from conservative management to surgery [6, 13, 27]. Most of the recent large series highlight that the goals of surgical treatment are correction of spinal deformity and stabilization of spinal instability [6, 13, 27]. As recommended, we performed a single stage posterior four-rod spinopelvic fixation, which was well maintained at a 5-year follow-up examination. In addition, ligation and transection of the dural sac and cauda equina stopped the CSF leakage and no other related adverse neurological symptoms were observed during the follow-up examinations.

In conclusion, to our knowledge, this is the first reported case of CSA associated with CSF-cutaneous fistula. We believe that disruption of the intact spinal muscles and skin overlying the dural tear site led to CSF leakage, consequently leading to a CSF-cutaneous fistula at the level of L5 post-traumatic CSA.

We recommend ligation and transection of the dural sac and posterior four-rod spinopelvic fixation for a successful treatment of post-traumatic CSA with CSF-cutaneous fistula.

## Supplementary information

**Additional file 1.** Timeline.

## Data Availability

All data generated or analyzed during this study are included in this article.

## Abbreviations

CSA: Charcot spinal arthropathy
CSF: Cerebrospinal fluid
CT: Computed tomography
MRI: Magnetic resonance imaging
