# Supplementary material for: Cerebrospinal fluid-cutaneous fistula associated with post-traumatic Charcot spinal arthropathy: a case report and review of literature
Source: BMC Musculoskelet Disord. 2020 Jun 29;21:412. doi: 10.1186/s12891-020-03451-7 (PMC7325221; doi:10.1186/s12891-020-03451-7)
Supplement: Supplementary file 1 — Additional file 1. Timeline. [file 12891_2020_3451_MOESM1_ESM.docx]

| **Dates** | **Summaries from initial and**  **follow-up visits** | **Diagnosis** | **Surgery** |
| --- | --- | --- | --- |
| 01/2006 | Initial visit to other hospital (Motorcycle accident) | T11 burst fracture with spinal cord injury | D9-L2 posterior fixation at other hospital |
| 01/24/2013 | initial visit to  plastic surgeon | Pressure sore on back | Debridement and gluteus maximus myocutaneous flap coverage |
| 02/22/2013 | follow up visit to  plastic surgeon | Pressure sore on back | Debridement and local flap coverage |
| 03/20/2013 | Consultation to  colorectal surgeon | Wound-anus fistula | Temporary colostomy |
| 04/04/2013 | follow up visit to  plastic surgeon | Pressure sore on back | Debridement and rotational advanced gluteus maximus musculocutaneous flap coverage |
| 05/06/2013 | follow up visit to  plastic surgeon | Pressure sore on back | Debridement & irrigation |
| 05/20/2013 | follow up visit to  plastic surgeon | Pressure sore on back | Debridement and bilateral rotational flap coverage |
| 08/12/2013 | follow up visit to colorectal surgeon | Wound-anus fistula | Colostomy repair |
| 10/23/2013~ 11/13/2013 | follow up visit to  plastic surgeon | Pressure sore on back | Debridement and irrigation every day |
| 11/18/2013 | follow up visit to  plastic surgeon | Pressure sore on back | Debridement and myocutaneous flap coverage and skin graft |
| 01/15/2014 | Consultation to orthopaedic surgeon | CSF-cutaneous fistula associated with CSA |  |
| 02/04/2014 | follow up visit to orthopaedic surgeon | CSF-cutaneous fistula associated with CSA | transection of the dural sac and cauda equina/  posterior four-rod spinopelvic fixation. |
| 05/05/2015 | Visit to orthopaedic surgeon | CSF-cutaneous fistula associated with CSA | Removal of right iliac screw |
| 06/11/2019 | Visit to orthopaedic surgeon (postoperative 5years) | CSF-cutaneous fistula associated with CSA |  |

**Abbreviations**

CSA, Charcot spinal arthropathy

CSF, cerebrospinal fluid
